# Billroth on Removal of Cancer

**Published:** 1882-08

**Authors:** 


### Billroth on Removal of Cancer.

In the course of a letter from Billroth to Dr. Wywodzew, concerning the nature of the last illness of Pirogoff (a translation of which appeared in the Boston *Medical and Surgical Journal*), this great surgeon, discussing the propriety of operative interference for the relief of cancer of the jaw, from which Pirogoff was suffering, says: "Interesting and instructive as the results of the microscopic examination are in such cases, and the etiology of the development anatomically so well illustrated, the diagnois of cancer in this case would not have influenced me to an operation. A man over seventy, of active mental habits, yet showing all the signs of bodily marasmus, with a cataract in each eye, etc., has no possible chance of withstanding an operation such as I would have been compelled to make, even in order to prevent a recurrence for a very short period of time. My experience, of thirty years, as a surgeon, has taught me that those sarcomata and carcinomata which originate away back on the upper jaw can never be radically removed by operative interference, when one operates so as to provide for the probability of the patient surviving the operation. One is so disturbed in this region, partly by technicalities, part anatomically, that a true and total extirpation cannot be made, save in those isolated cases where one has to do with an encapsulated tumor. I am no longer the bold, unterrified operator I used to be."

